# Nicotinamide restores tissue NAD^+^ and improves survival in rodent models of cardiac arrest

**DOI:** 10.1371/journal.pone.0291598

**Published:** 2023-09-15

**Authors:** Xiangdong Zhu, Jing Li, Huashan Wang, Filip M. Gasior, Chunpei Lee, Shaoxia Lin, Cody N. Justice, J. Michael O’Donnell, Terry L. Vanden Hoek

**Affiliations:** 1 Center for Advanced Resuscitation Medicine and Department of Emergency Medicine, Center for Cardiovascular Research, University of Illinois Hospital & Health Sciences System, Chicago, Illinois, United States of America; 2 Department of Physiology and Biophysics, Center for Cardiovascular Research, University of Illinois Hospital & Health Sciences System, Chicago, Illinois, United States of America; Nippon Medical School Graduate School of Medicine, JAPAN

## Abstract

Metabolic suppression in the ischemic heart is characterized by reduced levels of NAD^+^ and ATP. Since NAD^+^ is required for most metabolic processes that generate ATP, we hypothesized that nicotinamide restores ischemic tissue NAD^+^ and improves cardiac function in cardiomyocytes and isolated hearts, and enhances survival in a mouse model of cardiac arrest. Mouse cardiomyocytes were exposed to 30 min simulated ischemia and 90 min reperfusion. NAD^+^ content dropped 40% by the end of ischemia compared to pre-ischemia. Treatment with 100 μM nicotinamide (NAM) at the start of reperfusion completely restored the cellular level of NAD^+^ at 15 min of reperfusion. This rescue of NAD^+^ depletion was associated with improved contractile recovery as early as 10 min post-reperfusion. In a mouse model of cardiac arrest, 100 mg/kg NAM administered IV immediately after cardiopulmonary resuscitation resulted in 100% survival at 4 h as compared to 50% in the saline group. In an isolated rat heart model, the effect of NAM on cardiac function was measured for 20 min following 18 min global ischemia. Rate pressure product was reduced by 26% in the control group following arrest. Cardiac contractile function was completely recovered with NAM treatment given at the start of reperfusion. NAM restored tissue NAD^+^ and enhanced production of lactate and ATP, while reducing glucose diversion to sorbitol in the heart. We conclude that NAM can rapidly restore cardiac NAD^+^ following ischemia and enhance glycolysis and contractile recovery, with improved survival in a mouse model of cardiac arrest.

## Introduction

Sudden cardiac arrest (CA) is a leading cause of death that is related to global ischemia/reperfusion (I/R) injury of the heart and brain. While the return of spontaneous circulation (ROSC) is achieved in 30% of patients with CA, the rate of survival to hospital discharge is only 8.8% [1]. Post-resuscitation myocardial dysfunction contributes to the low survival rate after ROSC. Deaths within the first day following ROSC are typically due to myocardial stuning and injury [2]. However, no drugs are currently available to enhance CA survival to hospital discharge [3]. The issue of impaired cardiomyocyte bioenergetics is a significant concern in CA that has yet to be adequately addressed. Myocardial functional recovery and higher mean arterial pressure after ROSC within the first few hours are related to survival at discharge and neurologic outcomes [4]. Given the role of nicotinamide (NAM) in bioenergetics and as a potential metabolic treatment, we focused on its role in NAD^+^ homeostasis, glycolysis, and glucose diversion into sorbitol after cardiac arrest. In addition, its potential role during acute global I/R may be different from other chronic conditions such as neurodegenerative diseases in which NAD^+^ deficiency has been studied [5].

NAD^+^ plays a significant role as a coenzyme for hydride transfer enzymes that participate in both glycolytic and mitochondrial ATP synthesis. NAD^+^ serves as a precursor for NADPH, which is necessary for anabolic pathways and the detoxification of reactive oxygen species during reperfusion. Furthermore, NAD^+^ is a vital substrate for enzymes such as sirtuins and poly(ADP-ribose) polymerases (PARP), which play a crucial role in DNA damage responses. Robust activation of PARP-1 by oxidative stress depletes myocardial NAD^+^ stores during reperfusion [6], and NAD^+^ depletion has been implicated as a major cause of necrotic cell death of cardiomyocytes [7]. The opening of the mitochondrial permeability transition pore during reperfusion results in the depletion of mitochondrial NAD^+^, leading to a decline in ATP production and eventual cell death [8].

Under normal physiological conditions, biosynthetic pathways rely on dietary sources of tryptophan and NAD^+^ precursors such as NAM, nicotinic acid, and nicotinamide riboside to compensate for the consumption of NAD^+^ by signaling enzymes. However, in pathological conditions, the availability of dietary precursors may be insufficient to maintain adequate NAD^+^ levels, thus requiring supplementation with either NAD^+^ or its precursors. NAD^+^ supplementation has been shown to protect H9C2 cardiomyocytes against hypoxia/reoxygenation injury [9]. However, the acute effects of boosting cellular NAD^+^ level on metabolic pathways are poorly understood, and the effect of NAD^+^ repletion on cardiac function and outcome of cardiac arrest are not well studied. We hypothesized that NAD^+^ supplementation promotes glycolysis and mitigates glucose diversion into sorbitol in heart following I/R injury, leading to enhanced contractile recovery and survival after cardiac arrest.

This study aimed to assess the impact of nicotinamide administration on the contractile recovery of mouse cardiomyocytes and isolated rat hearts during simulated global ischemia/reperfusion (I/R) injury, as well as its effect on survival in a mouse model of cardiac arrest. Several NAD⁺ precursors including niacin, NAM, nicotinamide mononucleotide (NMN) and nicotinamide riboside were used in studies to generate more cellular NAD⁺. NAM was chosen in this study because it does not cause flushing and is better tolerated than niacin [10]. NAM is also known to inhibit PARP-1, a NAD consuming enzyme that is activated by reperfusion injury [6]. On the other hand, the direct absorption of NAD^+^ by cells is limited due to its large polarity [11]. In this study, we found that supplementation with NAM rapidly restored cardiac tissue NAD^+^ content, enhanced glycolysis and ATP generation while reducing glucose shunting to sorbitol, promoted recovery of contractile function and improved mouse survival after cardiac arrest. In addition, it also decreased release of nicotinamide phosphoribosyltransferase (NAMPT) into plasma, a critical rate-limiting enzyme that regulates intracellular NAD^+^ production.

## Materials and methods

### Ethics statement

The study adheres to the National Institutes of Health’s (NIH) publication No. 85–23, 2011 8^th^ edition, which outlines the Guide for the Care and Use of Laboratory Animals. The University of Illinois at Chicago’s Institutional Animal Care and Use Committee approved the procedures involving the mouse model of cardiac arrest, Langendorff model of rat heart perfusion, and isolation of neonatal mouse cardiomyocytes.

### Materials

NAM, sorbitol and sorbitol dehydrogenase were purchase from Sigma (St Louis, MO). The NAD/NADH cell based assay kit was purchased from Cayman (Ann Arbor, MI). The ELISA kit for NAMPT was purchased from Adipogen (San Diego, CA). The ATP colorimetric assay kit and lactate assay kit were purchased from Abcam (Boston, MA).

### Primary culture of mouse cardiomyocytes and measurement of contractile velocity

Primary cultures of mouse ventricular cardiomyocytes were prepared from hearts of 1 to 2-day-old neonatal C57BL6/J mice (Jackson, Bar Harbor, ME) as described previously [12]. Neonatal mice were sacrificed by cervical dislocation without presedation. To isolate the cells, 1 to 2-day-old neonatal C57BL6/J mouse hearts were excised and exposed to 0.1% trypsin in Hanks’ balanced salt solution (HBSS) without Ca^2+^ and Mg^2+^ (pH 7.4) for 8 minutes at 37°C. After discarding the first cell suspension, subsequent suspensions were added to trypsin inhibitor solution in cold HBSS with Ca^2+^ and Mg^2+^ (pH 7.4) for 5 to 6 cycles until all cardiac cells were isolated. To remove fibroblasts, the isolated cells were pre-plated for 90 minutes at 37°C. The supernatants obtained were then centrifuged and plated at a density of 0.6 × 10^6^ on laminin-coated coverslips using MEM supplemented with 10% fetal bovine serum (Invitrogen, Carlsbad, CA), 50 U/ml penicillin, and 1.5 μM B12 (Sigma, St. Louis, MO). The purity of myocytes was determined to be around 90% via immunofluorescent staining for α-sarcomeric actin and myosin heavy chain (Sigma, St. Louis, MO). Experiments were conducted on 6–8 day cultures.

Synchronously contracting cells on glass coverslips were transferred to a 1.2 ml Sykes-Moore perfusion chamber and exposed to a simulated ischemia/reperfusion (I/R) protocol as previously described [13]. After a 30-minute equilibration period, the cardiomyocytes were exposed to simulated ischemia for 30 minutes, followed by 90 minutes of reperfusion, and then treated with isoproterenol (10 μM) for 10 minutes. This cell model of myocardial stunning has been established by our group and tested with hypothermia and lipid emulsion treatment [14]. Two experimental groups were examined including control and NAM treatment. NAM was added at the start of reperfusion. The contractile velocity of cardiomyocytes was measured by speckle image velocimetry [14]. The movement of cardiomyocytes was monitored using phase-contrast microscopy following the same field of cells over time. One field of cardiomyocytes (~500 cells) was selected for each experiment, and eight-second videos were acquired at predetermined time points using phase-contrast optics (20X magnification, 33 frames per second). To quantify contraction velocity, Speckle Image Velocimetry was employed with custom MATLAB software (Mathworks, Natick, MA). Time points selected for contractility measurement are Is5, R10, R60 and R90, which thoroughly assess the contractility changes during ischemia (the time cells stopped beating), early and later phases of reperfusion, and the end of reperfusion.

### Mouse model of cardiac arrest

The procedures for the mouse model of cardiac arrest have been described previously [15,16]. In brief, adult female C57BL/6 mice were anesthetized with 100 mg/kg of ketamine and 10 mg/kg xylazine. Additional analgesic dose may be given as needed. Mice were intubated, ventilated and cannulated. The mean arterial blood pressure (MAP) was measured with a polyethylene (PE-10) catheter inserted into the carotid artery. Following 20 min stabilization with MAP > 80 mmHg and a partial pressure of end-tidal CO_2_ (P_ETCO2_) > 35 mmHg, asystolic cardiac arrest was then induced by i.v. administration of 0.08 mg/g potassium chloride solution. After the 10-minute arrest, chest compressions, mechanical ventilation, and scheduled fluid administration were used to attempt cardiopulmonary resuscitation (CPR). Mice that were successfully resuscitated were then hemodynamically monitored on mechanical ventilation for up to 4 hours. Immediately upon ROSC, a total of 20 mice were randomized into two groups to receive NAM or normal saline (NS) given intravenously (n = 10 per group), 4 h survival was assessed. The surviving mice at the end of experiment were euthanized by manual cervical dislocation under anesthesia and all efforts were made to minimize suffering.

### Measurement of cellular and tissue NAD^+^ concentrations

The intracellular concentration of NAD^+^ was determined with an NAD^+^/NADH quantification kit (Cayman Chemical, Ann Arbor, Michigan) following the manufacturer’s instructions. To measure NAD^+^ content in cardiomyocytes, cells (1 × 10^6^) were washed and lysed in 110 μl of permeabilization buffer for 30 min. Cell lysates were collected into a microcentrifuge tube, followed by centrifugation at 11,000 g for 1 min, and supernatants were saved for assay. To measure NAD^+^ in heart tissues, tissues lysates were extracted with 100 μl of 0.5 M perchloric acid for 30 min, and then neutralized with 100 μl of 0.55 M K_2_CO_3_. The neutralized extracts were centrifuged at 10,000 g for 1 min and supernatants were used for NAD^+^ assay.

To detect NAD^+^ content, the extracted supernatant (100 μL) was transferred into a 96-well plate, incubated with 100 μL of reaction solution for 90 min and measured at 450 nm with a microplate reader (Thermo scientific). NAD^+^ concentrations were calculated according to an NAD^+^ standard curve and were expressed as nM for cardiomyocytes and nMol/g protein for tissue lysates.

### Measurement of sorbitol content

Sorbitol accumulation during I/R can contribute to tissue injury and requires NAD^+^ for its clearance. In additional experiments, a total of 10 mice were randomized into saline and NAM treatment group (n = 5 per group), subjected to KCl induced cardiac arrest and resuscitation as above. Mice were sacrificed under anesthesia 30 min after ROSC and mouse hearts were harvested for the measurement of sorbitol content. In preliminary studies, we found R30 min is the peak time for sorbitol accumulation. Protein from heart tissue lysates was precipitated by adding 25 μl of 0.3 M ZnSO_4_ solution to 150 μL of lysates, followed by neutralization with 25 μL of 0.475 M NaOH [17]. The supernatant was separated by a 10 min centrifugation at 5,000 g at 4°C. 100 μl of the supernatant was incubated with 50 μL of 0.15 M Tris buffer (pH 8.6) containing D-sorbitol dehydrogenase (2 U/mL), 3 mM NAD^+^ and 10 mM EDTA for 30 min at 37°C. The absorbance of NADH production was measured at 340 nm by a spectrophotometer [18]. Sorbitol standards ranging from 15.7 to 500 μM were similarly analyzed to generate a standard curve. Sorbitol concentration was normalized with protein content in tissue lysates and expressed as μMol/g tissue lysates.

### Measurement of plasma NAMPT concentration

Extracellular NAMPT is a pro-inflammatory cytokine associated with I/R injury and its release may be affected by NAD^+^ homeostasis. Plasma NAMPT concentration at the end of experiments was measured by NAMPT ELISA kit per manufacturer’s instruction (Adipogen, San Diego, CA). Previously we found R4h is the optimal time to determine the treatment effect on eNAMPT (PBEF) concentration [19].

### Isolated rat heart ischemia / reperfusion model

Adult male Sprague**-**Dawley rats were heparinized (1000 IU) and anaesthetized (100 mg/kg sodium pentobarbital i.p.), hearts excised and retrograde perfused in the Langendorff model as described in detail previously [20,21]. Briefly, hearts were excised into ice-cold perfusion fluid, the aorta cannulated and coronary vessels perfused at a hydrostatic pressure of 90 cm H_2_O with modified Krebs-Henseleit buffer containing (in mMol/L): 116 NaCl, 4 KCl, 1.5 CaCl_2_, 1.2 MgSO_4_ and 1.2 NaH_2_PO_4_, equilibrated with 95% O_2_/5% CO_2_ with 0.4 mMol/L oleate/albumin complex (3:1) and 10 mM glucose [22,23]. A water-filled, latex balloon in the left ventricle set to a diastolic pressure of 5–10 mmHg provided hemodynamic recordings (Powerlab, AD Instruments, Colorado Springs, CO). Hearts were maintained at 37°C. Isolated rat hearts were retrograde perfused for 20 minutes. Perfusate flow was then stopped for an ischemic period of 18 minutes. This ischemic period- chosen in these experiments is based on preliminary studies showing that contraction of the rat heart could be restarted during reperfusion without arrhythmia and myocardial necrosis. Following the ischemic insult, perfusate flow was reestablished. After a 20- minute reperfusion period, hearts were freeze- clamped in liquid nitrogen and stored at -80°C. The ex vivo rat heart I/R perfusion protocol was used for the control group and 1 mM nicotinamide treatment group started at the time of reperfusion. Previous studies have found that 1 mM NMN increased cellular NAD^+^ and glycolysis in the Langendorff model of heart perfusion [24], therefore NMN was also used in some additional studies and served as a control. Peak systolic, diastolic and developed pressures, heart rate, and rates of contraction and relaxation over time (dP/dt) were documented. The rate-pressure product (RPP) was the product of the LVDP and the heart rate in beats per min. NAD^+^, lactate, ATP and sorbitol in heart tissues were quantified after perchloric acid extraction. Lactate and ATP quantity in heart tissues were measured using colorimetric assay kits (Abcam, Cambridge, MA).

### Statistical analysis

Results were expressed as means ± SD. For comparison among the different treatment groups, one-way ANOVA were used with post hoc examination by Fisher’s least significant difference test. Kaplan-Meier survival analysis was performed using the Breslow test. T-tests were used for contractile function of isolated hearts. *P* < 0.05 was considered statistically significant.

## Results

### NAM supplementation restored intracellular NAD^+^ content and improved the recovery of contractility in mouse cardiomyocytes exposed to I/R

**Fig 1:**
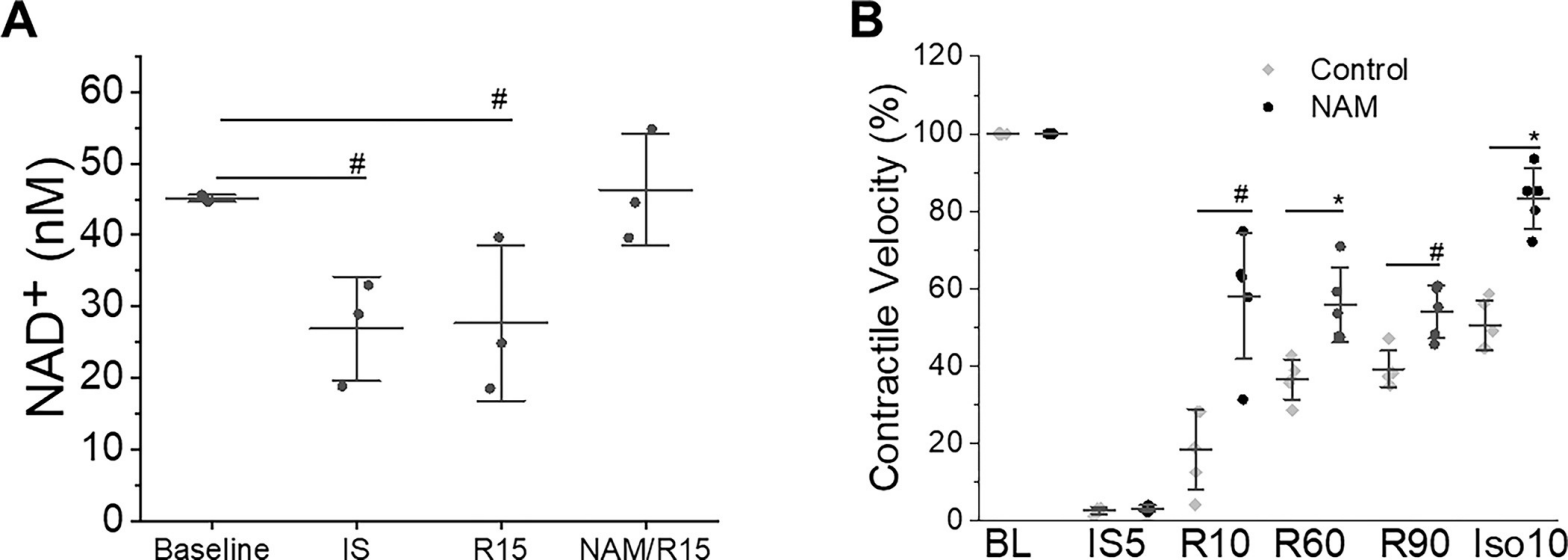
**A. NAM restores NAD**^**+**^ **content in cardiomyocytes after I/R injury.** Cardiomyocytes were subjected to 30 min simulated ischemia and 15 min reperfusion, and NAM (100 μM) was added at the start of reperfusion. NAD^+^ content in cardiomyocytes was measured using a cell-based assay kit, N = 3 for each group. **B. Effect of NAM on contractile recovery of cardiomyocyte after I/R injury.** Cells were subjected to 30 min ischemia and 90 min reperfusion, followed by 10 min isoproterenol (10 μM). NAM (100 μM) was added at the start of reperfusion. Speckle Image Velocimetry was used to quantify contraction velocity with custom written MATLAB software. N = 5 for each group. BL, baseline. #, *P*<0.05 and *, *P*<0.01. Data are presented as means ± SD.

To investigate the potential benefits of NAM, we examined whether it could increase the intracellular NAD^+^ content in cardiomyocytes and improve the recovery of contractile function following simulated I/R. NAM has previously been shown to serve as a precursor for NAD^+^ biosynthesis through the salvage pathway in mammalian cells [25]. NAM at mM concentrations has been used for cardiomyocyte survival after prolonged hypoxia/reoxygenation injury [26,27]. We first tested the effects of NAM on NAD^+^ recovery in mouse cardiomyocytes subjected to 30 min simulated ischemia followed by 15 min reperfusion [14]. As this model of cardiomyocyte stunning is less severe and there is no cell death involved, lower concentration of NAM was chosen for this study. NAM at100 μM was added at the start of reperfusion. NAD^+^ content was decreased from 45.1 ± 0.5 nM before ischemia to 26.9 ± 7.3 nM at the end of 30 min ischemia. Treatment with NAM completely restored the cellular level of NAD^+^ at 15 min of reperfusion (46.3 ± 7.7 nM), while NAD^+^ in untreated cells remained significantly depleted compared to baseline conditions (27.7 ± 10.9 nM, *P*<0.05, Fig 1A). This rescue of NAD^+^ depletion was associated with a significant difference in functional contractile recovery during reperfusion with peak at 10 min reperfusion (58.1 ± 16.2% return of baseline contractile velocity vs.18.5 ± 10.4% in control cells, n = 5, *P*<0.05), with enhanced contractile reserve seen after isoproterenol treatment (83.3 ± 7.8% vs. 50.5 ± 6.5% in control cells. *P*<0.01, Fig 1B).

### NAM increased mouse survival after cardiac arrest

**Fig 2:**
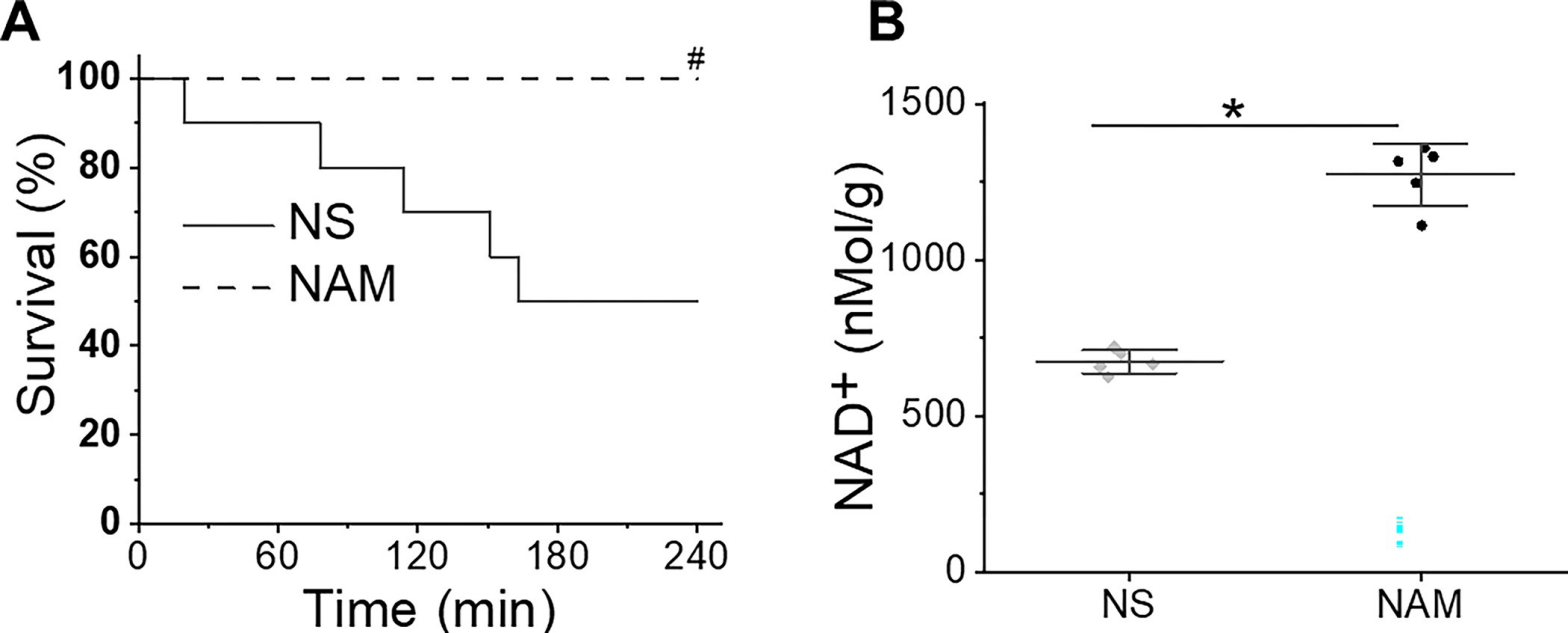
**A. NAM increased mouse survival after cardiac arrest.** Mice were subjected to 10 min cardiac arrest followed by CPR, and randomly assigned to receive either saline or 100 mg/kg NAM immediately after return of spontaneous circulation (ROSC). Improved mouse survival seen by Kaplan -Meyer analysis (N = 10 in each group). **B. NAM increased heart tissue NAD**^**+**^ **content.** Mouse hearts were obtained at 4 h after ROSC and heart tissue NAD^+^ content was measured by a colorimetric assay kit and normalized to soluble protein (N = 5 in each group). #*P* <0.05 and *P<0.01. Data are presented as means ± SD.

We next tested the effect of NAM administration during CPR on 4 h mouse CA survival. In diabetes studies in rodent NAM has been used in a daily dose of 100–500 mg/kg [28,29]. Our preliminary data showed that two doses of NAM (100 mg/kg and 500 mg/kg) for treatment have a similar effect on cardiac arrest survival. Therefore, we selected a lower dose of NAM (100 mg/kg) for subsequent experiments. As seen in Fig 2, NAM administered IV immediately after ROSC resulted in improved mouse survival, with 10/10 survival at 4 h as compared to 5/10 in the NS group (*P*<0.05). Other parameters at both baseline and resuscitation were similar in both groups (Table 1). Compared to saline treated mice, NAM-treated mice displayed an improved NAD^+^ content in hearts obtained at 4 h post-ROSC (1272.5 ± 99.9 nMol/g vs. 674.4 ± 37.6 nMol/g saline control, *P*<0.01).

**Table 1:** Characteristics at baseline, CPR period and 4h after ROSC in NS and NAM groups.

		Control (n = 10)	NAM (n = 10)
**Baseline**	Weight (g)	28.9 ± 1.9	29.4 ± 3.5
	MAP (mmHg)	87.1 ± 8.7	88.6 ± 8.7
	ETCO2 (mmHg)	41.1 ± 2.5	40.2 ± 2.1
	Heart rate (bpm)	274.3 ± 41.4	281.7 ± 28.7
**Resuscitation**	CPR time to ROSC (s)	130.2 ± 18.7	120.8 ± 10.6
**R4h**	MAP (mmHg)	51.4 ± 8.6	59.4 ± 10.9
	ETCO2 (mmHg)	33.2 ± 1.6	33.3 ± 0.9
	Heart rate (bpm)	481.2 ± 74.1	458.4 ± 111.3
	Survival (%)	5 (50%)	10 (100%)^#^

#, *P*<0.05, significant difference from saline treated control mice.

### NAM prevented sorbitol accumulation in heart tissues and decreased eNAMPT concentration in mouse blood

**Fig 3:**
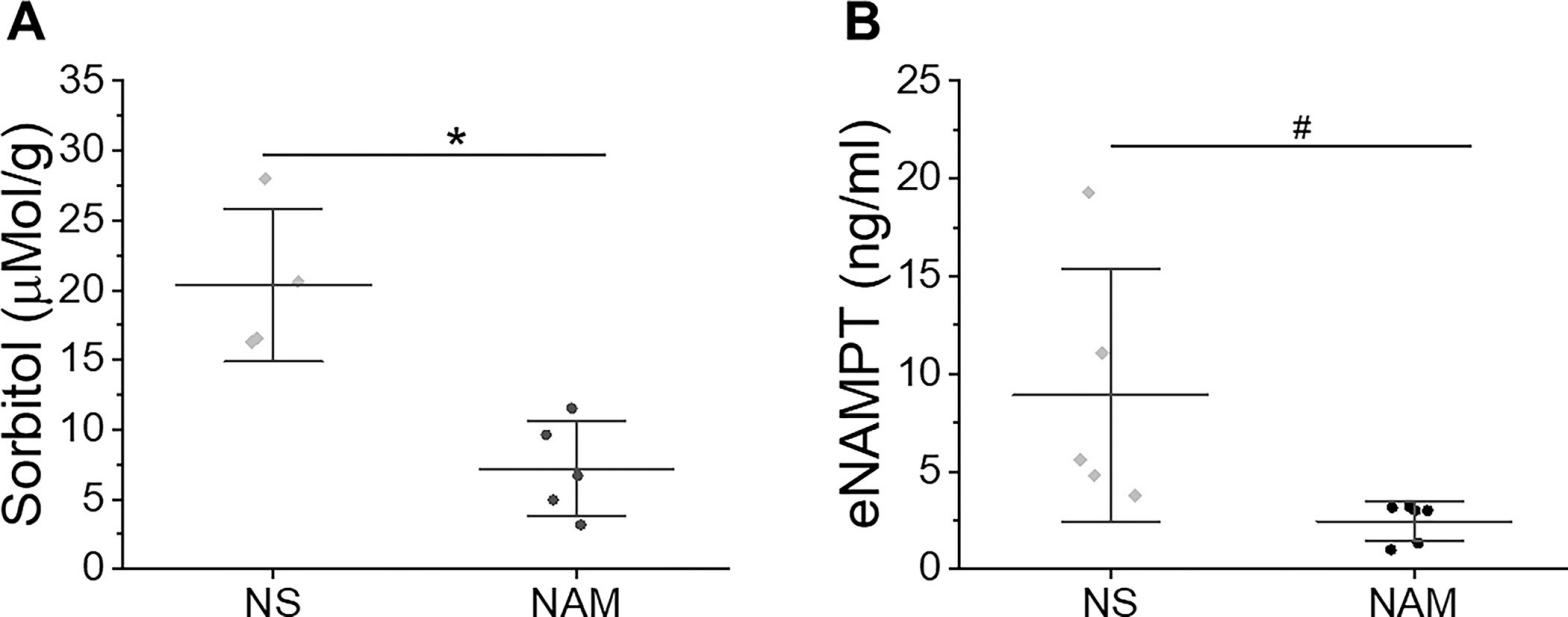
**A. NAM reduced sorbitol accumulation in heart.** Mice were subjected to 10 min asystolic arrest followed by resuscitation. Sorbitol content in hearts at 30 min reperfusion was measured. N = 4 for saline control (one mouse was omitted from the study due to surgical failure) and N = 5 for NAM groups. **B. NAM reduced NAMPT release into plasma (eNAMPT).** Mice were subjected to 10 min asystolic arrest followed by resuscitation. Plasma NAMPT concentrations were measured at 4 h after ROSC by ELISA. N = 5 for saline control and N = 6 for NAM groups. #, *P*<0.05 and *, *P*<0.01. Data are presented as means ± SD.

The polyol pathway, a bypass pathway of glucose metabolism initiated by aldose reductase, has been shown to play an important role in mediating tissue I/R injury [30]. As NAD^+^ is a coenzyme for sorbitol dehydrogenase and substrate for GAPDH, we tested whether NAM supplementation decreased the tissue sorbitol content during early reperfusion. As shown in Fig 3, NAM treatment decreased sorbitol concentration in heart tissues from 20.4 ± 5.4 μMol/g for saline-treated control to 7.2 ± 3.4 μMol/g (*P*<0.01) at 30 min of reperfusion (Fig 3A).

Several metabolic and inflammatory disorders, such as cardiac arrest, have been linked to eNAMPT levels [19,31,32]. We examined whether NAM supplementation is associated with decreased eNAMPT concentrations in plasma. eNAMPT could be detected 30 min after reperfusion and peaked at 4h post-ROSC. As shown in Fig 3B, plasma eNAMPT concentrations in NAM treated mice were significantly lower than control mice at 4 hours post-ROSC (2.4 ± 1.0 ng/ml vs. 8.9 ± 6.5 ng/ml, *P*<0.05).

### Langendorff rat heart model of global I/R

**Fig 4:**
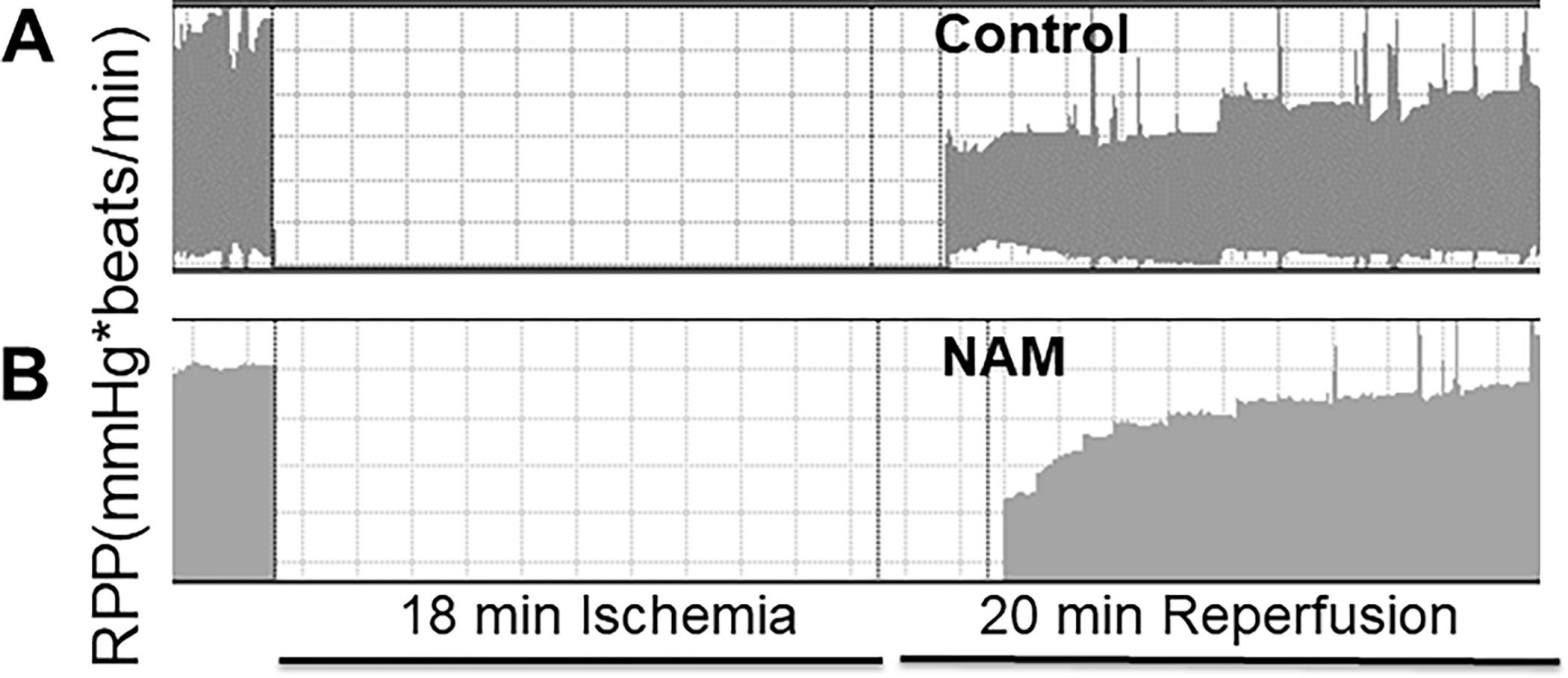
NAM restored the rate-pressure product (heart rate · LVDP) from isolated perfused hearts at 20 min of reperfusion. Rat hearts were subjected to 18 min no-flow global ischemia followed by 20 min reperfusion. Representative rate-pressure product tracing in buffer treated control hearts (A, N  =  4) and NAM (B, N  =  4) treated hearts. NAM was given at the start of reperfusion. Data are presented as means ± SD. #, *P*<0.05.

To determine the direct effect of NAM on heart, we used an isolated rat heart model to measure recovery of cardiac function from an ischemic insult. Fig 4A and 4B showed a representative tracing of cardiac function of buffer control and NAM treated hearts, respectively. Cardiac function was reduced by 26% following an 18 min ischemic insult in buffer-treated control hearts (Table 2, prearrest RPP 20,113 ± 310 mmHg × beats/min; post-arrest 14,816 ± 1299, *P* < 0.05), consistent with stunning. A significant reduction of left ventricular pressure (LVP), left ventricle developed pressure (LVDP) and–dP/dt was also observed after 20 minutes reperfusion (R20). NAM at 1 mM administration resulted in complete cardiac functional recovery following the ischemic insult, relative to preischemic function (prearrest RPP 21,460 ± 1520; postarrest 20,218 ± 1623). Compared to buffer treated control hearts, NAM treated hearts showed a better recovery of RPP at 20 min reperfusion (Table 2). However, other parameters, such as LVDP, LVP and–dP/dt showed no differences at R20 min between buffer and NAM treated hearts.

**Table 2:** Contractile parameters from pre-ischemic isolated hearts and at 20-min reperfusion following an 18-min ischemic insult.

Parameters	Control (*n* = 4)	NAM (*n* = 4)
***Pre-ischemic***		
RPP (mmHg*beats/min)	20,113 ± 310	21,460 ± 1520
LVDP (mmHg)	120 ± 9	105 ± 6
Heart rate (beats/min)	164 ± 13	192 ± 11
EDP (mmHg)	5 ± 1	7 ± 3
LVP (mmHg)	124 ± 9	112 ± 3
-dP/dt (mmHg/s)	-2.67 ± 0.55	-2.17 ± 0.30
+dP/dt (mmHg/s)	2.84 ± 0.74	2.69 ± 0.42
***Reperfusion* (*20 min*)**		
RPP (mmHg*beats/min)	14,816 ± 1299*	20,218 ± 1623^#^
LVDP (mmHg)	88 ± 14*	100 ± 15
Heart rate (beats/min)	161 ± 27	195 ± 32
EDP (mmHg)	7 ± 4	7 ± 1
LVP (mmHg)	94 ± 12*	106 ± 16
-dP/dt (mmHg/s)	-2.03 ± 0.53*	-1.95 ± 0.22
+dP/dt (mmHg/s)	2.16 ± 0.68	2.48 ± 0.52

*, *P*<0.05, significant difference between pre-ischemic Control vs. reperfused Control group.

#, *P*<0.05, significant difference between Control and NAM groups.

### Nicotinamide stimulates cardiac glycolysis and ATP production while reducing glucose shunting to the polyol pathway in an isolated rat heart model

**Fig 5:**
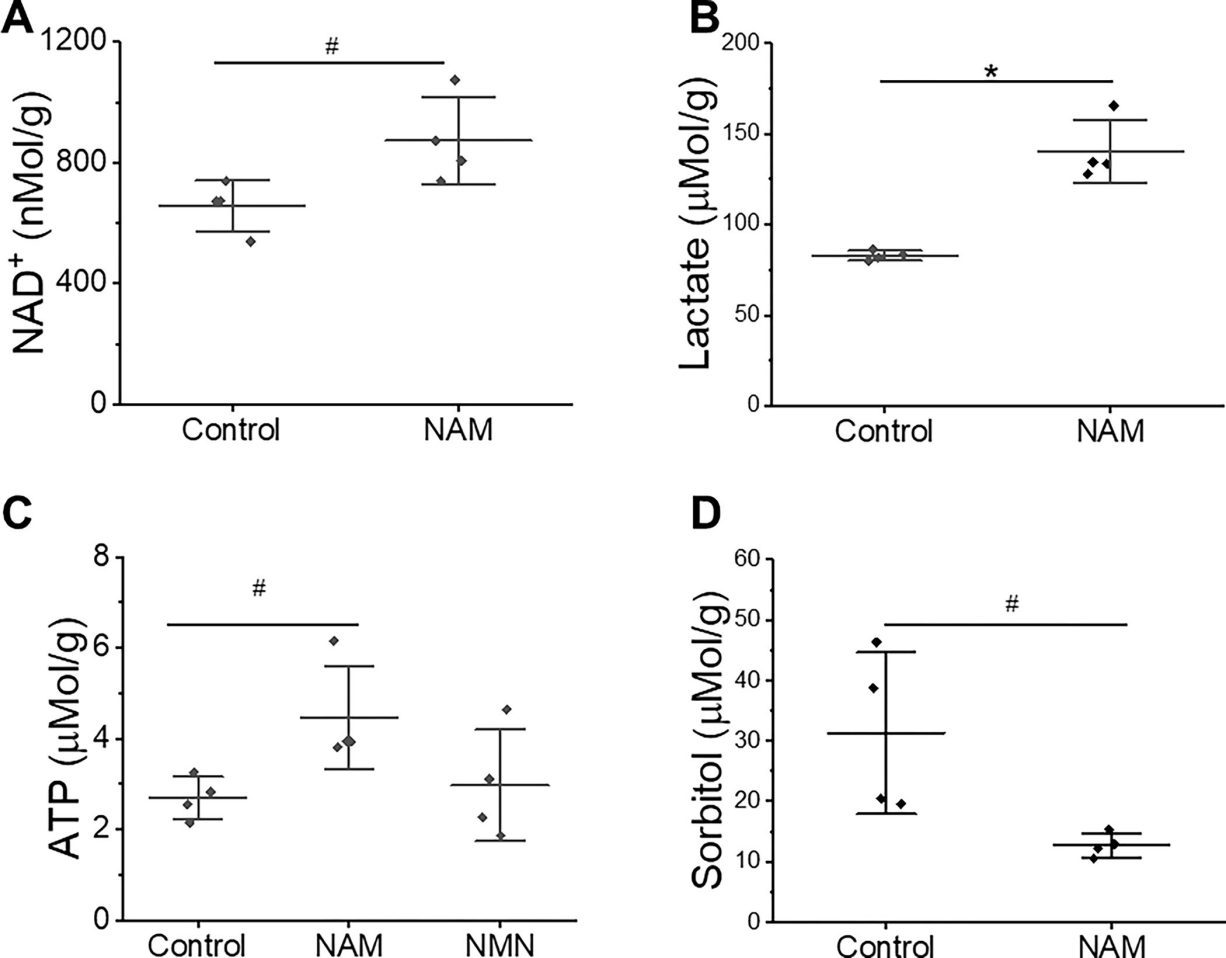
NAM enhanced NAD^+^, lactate and ATP production while reducing sorbitol concentrations in heart tissue. Rat hearts at 20 min of reperfusion were extracted and tissue levels of NAD^+^, lactate, ATP and sorbitol were measured. Data are presented as means ± SD. N  =  4 for control rat hearts and N  =  4 for NAM or NMN treated rat hearts. #, *P*<0.05 and *, *P*<0.01.

Since NAM is a precursor for NAD^+^, and NAD^+^ is a substrate for GAPDH in glycolysis, we hypothesized that NAM-reduced sorbitol content may be partly due to increased glycolysis and less shunting of glucose to the polyol pathway. Metabolic analysis showed that 1 mM of NAM added to perfused rat hearts caused a significant elevation of NAD^+^ from 656.6 ± 83.9 nMol/g to 873.3 ± 144.0 nMol/g (Fig 5A, *P*<0.05), lactate from 82.9 ± 2.7 μMol/g to 140.4 ± 17.1 μMol/g (Fig 5B, *P*<0.01), and ATP from 2.7 ± 0.5 μMol/g to 4.5 ± 1.1 μMol/g (Fig 5C, *P*<0.05), while decreasing sorbitol from 31.3 ± 13.4 μMol/g to 12.7 ± 2.0 μMol/g (Fig 5D, *P*<0.05) at R20 min. As a control, 1 mM NMN added at the start of reperfusion did not significantly enhance ATP production (Fig 5C).

## Discussion

The current study demonstrates that exogenous NAM supplementation confers remarkable cardioprotection against conditions of global I/R injury. NAM supplementation at the beginning of reperfusion enhanced contractile recovery both in mouse cardiomyocytes and isolated rat hearts subjected to simulated ischemia and reperfusion. In a mouse model of cardiac arrest, NAM supplementation enhanced 4- hour survival. These effects were attributed to replenishing tissue NAD^+^, which leads to enhanced glycolysis and reduced glucose shunting to sorbitol. These findings provide an insight into developing new strategies for the treatment of post-resuscitation myocardial dysfunction following cardiac arrest.

### NAM increases NAD^+^ content in the heart

NAD^+^ is a crucial cofactor involved in many enzymatic reactions of energy metabolism, such as glycolysis and oxidative phosphorylation. NAD^+^ levels are reduced in the myocardium at both ischemia and early reperfusion, and application of nicotinamide mononucleotide (NMN) 30 min before ischemia prevents the ischemia-induced decreases of NAD^+^ content in heart in a mouse model of myocardial infarction [33]. This study evaluates the effects of NAM on cardiac function and survival after cardiac arrest occurs, therefore NAM was added at the start of reperfusion rather than before ischemia. The results indicate that the addition of external NAM can enhance the levels of NAD^+^ within cells and stimulate the recovery of contractile function in both cardiomyocytes (Fig 1B) and isolated rat hearts (Fig 4). We chose the administration of NAM rather than NAD^+^ or NMN, because both NAD^+^ and NMN penetrate less efficiently than NAM through the plasma membrane [34,35]. We showed that exogenous NAM added at the start of reperfusion can restore intracellular NAD^+^ concentrations at R15, suggesting that NAM can be quickly utilized by cardiomyocytes. The effect of NAM on the recovery of cardiac contractile function is rapid and may not require changes in protein expression. Therefore, we focused our studies on the metabolic changes caused by NAM. Although cellular NAD^+^ was fully restored in stunned cardiomyocytes by NAM, the contractile velocity was only partially recovered, indicating other molecules beyond NAD^+^ may also participate in myocardial contractility.

### The effect of NAM is mediated through enhanced energy production

Several studies have linked glycolysis to cardioprotection. Glycolytic activity during the transition period from anaerobic to aerobic metabolism has been shown to be essential for heart functional recovery in both isolated perfused hearts [24,36] and cardiac arrest [19,37]. Enhanced aerobic glycolysis was linked to lower ROS levels and protected cardiomyocytes against ischemia-reperfusion injury [38]. Other studies found that transient acidosis conferred cardioprotection against myocardial I/R injury by activating the PI3K-AKT-eNOS pathway [39,40]. In this study lactate and ATP production were evaluated to reflect the glycolytic activity in the heart. Our results showed that NAM remarkably increased lactate and ATP production of heart tissues at 20 min of reperfusion (Fig 5B and 5C), which suggests that NAM promotes cardiac functional recovery by increasing glycolytic activity. By contrast, NMN given at reperfusion did not significantly enhance ATP levels in heart measured at R20 min (Fig 5C). The exact reason is unknown but may be related to the small sample size in this study or organ specific absorption. In a hemorrhagic shock model of ischemia and resuscitation, NMN enhanced ATP levels in kidney but not in liver [41]. Su et al. found that exogenous NAD^+^ attenuated myocardial and neurologic dysfunction in a rat model of cardiac arrest by improving mitochondrial complex I respiratory capacity, which involves the NAD^+^-Sirtuin3-NDUFA9 deacetylation [42]. Taken together with our data, NAD^+^ repletion improves heart functional recovery via both cytosolic glycolysis and based upon the work by others, mitochondrial respiration.

### The effect of NAM on sorbitol accumulation

The polyol pathway is a two-step process that converts glucose to fructose. Glucose is first converted to sorbitol through a reaction catalyzed by aldose reductase, which uses NADPH as a cofactor. Sorbitol is then oxidized to fructose by sorbitol dehydrogenase, which utilizes NAD^+^ as a cofactor. However, the utilization of NAD^+^ by sorbitol dehydrogenase in the second step of the pathway can lead to a reduction in the amount of NAD^+^ available for GAPDH, thus limiting the rate of glycolysis. This reduction in glycolysis can be particularly detrimental to the ischemic heart, as it relies on glycolysis to produce ATP. The glucose flux via polyol pathway during ischemia can cause a decrease in ATP levels and ultimately lead to myocardial injury. Furthermore, sorbitol accumulation in heart tissue contributes to osmotic imbalance, resulting in a compensatory reduction of other osmolytes important for cardioprotection [21]. Our results showed that NAM can decrease glucose shunting to sorbitol in heart tissues at the early phase of reperfusion, which results in reduced osmotic imbalance.

### Effect of NAM on eNAMPT release

Extracellular NAMPT is released during cardiac arrest [37] and is regarded as a proinflammatory cytokine [43]. eNAMPT induces IL-1β, IL-6, IL-10 and TNF-α secretion from peripheral blood mononuclear cells [44]. Furthermore, studies have indicated that eNAMPT has anti-apoptotic effects on neutrophils [45]. Various cell types, including adipocytes, hepatocytes, cardiomyocytes, neutrophils, and macrophages, have been observed to release NAMPT [43]. While the exact mechanisms of NAMPT release remain unknown, several studies have suggested that eNAMPT is exported through a "non-classical" secretory pathway [46,47]. Our results showed that NAM dramatically attenuated plasma NAMPT levels, which indicates that NAM is not only able to restore tissue NAD^+^ via intracellular NAMPT, but also block NAMPT secretion. Future studies will focus on the source of plasma NAMPT and how NAM affects non-classical secretion pathways in these cells.

Increasing intracellular NAD^+^ levels by using NAM has been proposed as a potential therapeutic strategy to ameliorate metabolic and neurodegenerative diseases [48,49]. We showed in this study that NAD^+^ supplementation enhanced the contractile functional recovery of heart via increased glycolysis, decreased glucose shunting to sorbitol, and reduced release of NAMPT, a key enzyme for NAD^+^ salvage. Therefore, NAD^+^ supplementation by NAM could be a novel therapeutic strategy for cardiac arrest.

## Supporting information

S1 Raw images Supporting information for Fig 4. (PDF)

S1 Data Supporting information for other Figs. (XLSX)
