# Supplementary figures and images for: Nicotinamide restores tissue NAD+ and improves survival in rodent models of cardiac arrest
Source: PLoS One. 2023 Sep 15;18(9):e0291598. doi: 10.1371/journal.pone.0291598 (PMC10503771; doi:10.1371/journal.pone.0291598)

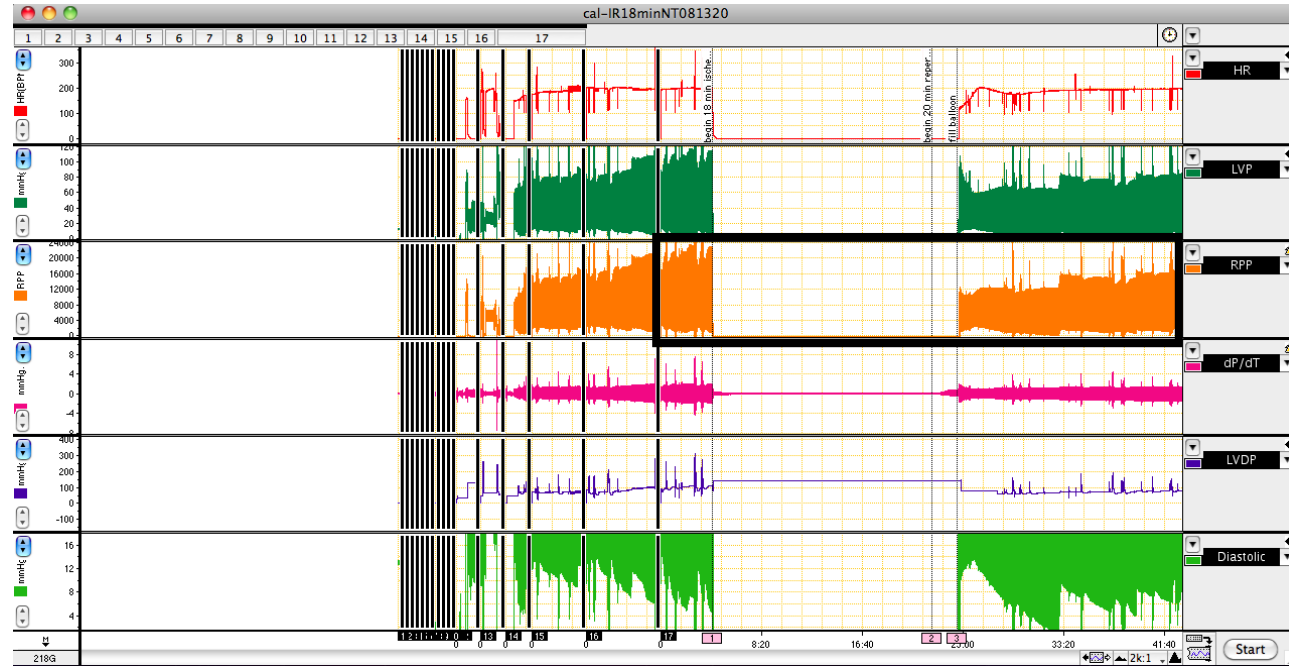

Control

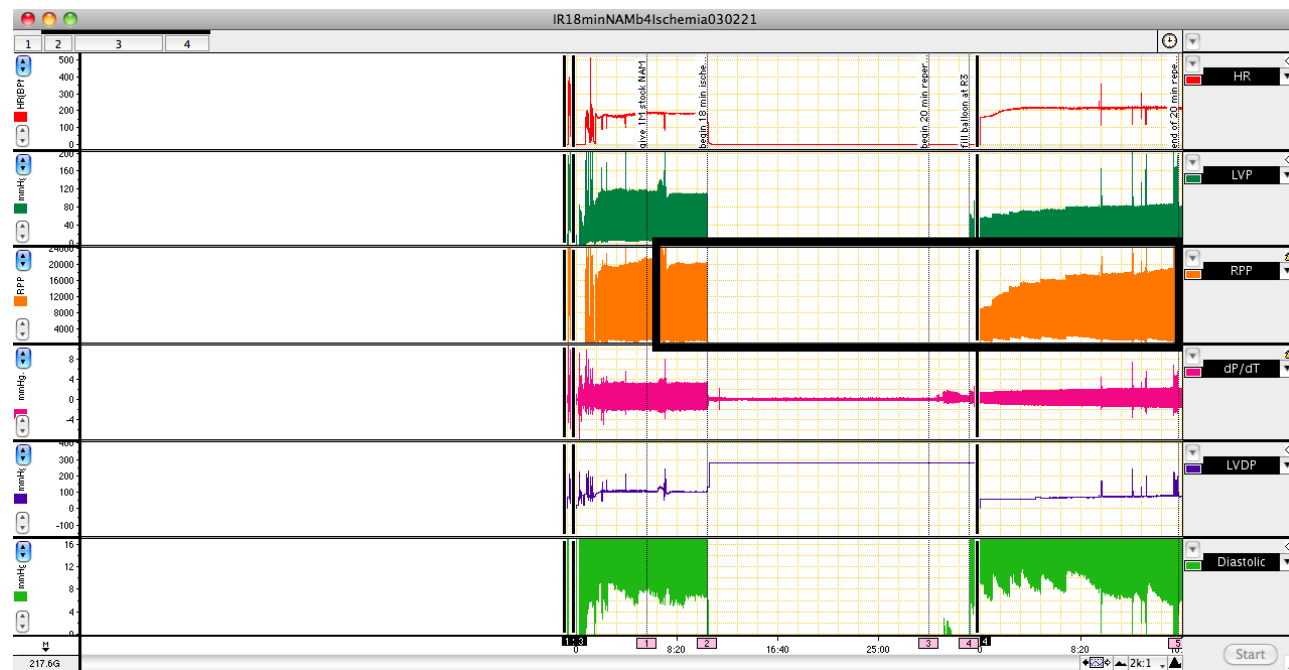

NAM

Supplement: S1 Raw images — (PDF) [file pone.0291598.s001.pdf]
